# Classifying High Strength Concrete Mix Design Methods Using Decision Trees

**DOI:** 10.3390/ma15051950

**Published:** 2022-03-06

**Authors:** Saleh J. Alghamdi

**Affiliations:** Department of Civil Engineering, College of Engineering, Taif University, P.O. Box 11099, Taif 21944, Saudi Arabia; sjalghamdi@tu.edu.sa

**Keywords:** mix design, high strength concrete, machine learning, compressive strength

## Abstract

Concrete mix design methods are used to determine proportions of concrete ingredients needed for certain workability and strength. Each mix design method operates under certain assumptions and suggests slightly different proportions. It is of great importance that site/construction engineers know the method by which the mix was designed. However, it can be difficult to know the designing method based solely on mix proportions. Hence, in this work, a decision trees model was used to classify high strength concrete mix design methods based on their produced concrete mix proportions. It was found that the trained decision tree model is capable of classifying mix design methods with high accuracy. Further, based on dimensionality reduction methods, the amount of cement in a concrete mix was found to be the paramount predictor of the used mix design method. In this work, a novel high-accuracy model for determining a mix design method based only on mix proportion is proposed.

## 1. Introduction

There are many methods for designing normal and high strength concrete mixes. The objective of designing a concrete mix is to determine the amounts of concrete mix constituents. Depending on how and where concrete is going to be used, its compressive strength and workability are determined, taking into account durability requirements. Normal strength mix design methods are used for designing concrete mixes, having strengths ranging from 20 to 55 MPa. Concrete having compressive strength higher than 55 MPa is considered to be high strength. For high strength concrete mix design, many methods can be used, including the American Institute of Concrete, ACI 211.4R-08, Guide for Selecting Proportions for High-Strength Concrete Using Portland Cement and Other Cementitious Materials [1], the Aïtcin method [2], and the modified department of environment method (Modified DOE) [3]. For a specified compressive strength, these methods suggest slightly different proportions of cement, water, sand, gravel, and chemical admixtures.

These methods are used by concrete manufacturers all over the world to design and make concrete mixes according to customers’ needs. It is of great importance that site/construction engineers know the method by which a concrete mix was designed, so that they can correctly adjust concrete mixes according to field conditions, interpret fresh concrete test results, as well as satisfy quality control requirements. However, determining the method by which a concrete mix was designed can be a difficult task, because design methods usually suggest similar mix proportions for required properties. In addition, to the best of the author’s knowledge, existing literature does not provide a solution to this problem. Hence, the objective of this work is to use machine learning models, in particular, decision trees to classify high strength concrete mix design methods based on their produced concrete mix proportions. The following section discusses the past and current implementations of machine learning algorithms in concrete technology.

### 1.1. Machine Learning Applied to Concrete Technology

At the present time, a considerable attention is directed towards machine learning and deep learning techniques, for their capability of solving complex problems. Some of these techniques were utilized to solve civil engineering problems including concrete mix design problems, such as the prediction of concrete’s 7, 14 and 28-day compressive strengths. Many researchers investigated the usefulness of using artificial neural networks (ANN) and linear regression models in the prediction of the strength of normal and high strength concrete [4,5,6,7,8,9,10], high-performance concrete [11,12,13], ultra-high-performance concrete [14], recycled aggregate concrete [15], structural lightweight concrete [16], bacterial concrete [17], green concrete [18], and self-consolidating concrete [19]. In addition to ANN, decision trees have also been used to predict compressive strength of different types of concrete including high strength and high-performance concrete [20,21], FRP-confined concrete [22], as well as recycled aggregate concrete [23,24]. In addition, ensemble methods were used to predict compressive strength of concretes containing fly ash [25], with higher accuracy than when decision tree models (DT) are used. Moreover, ANN, K-nearest neighbor (KNN) along with decision tree models, were leveraged to predict the healing performance of self-healing concrete [26]. Additionally, to evaluate the compressive strength of concrete mixes whose cement content is partially replaced with ceramic waste powder (CWP), ANN and decision tree models were leveraged. When the two models were compared, they demonstrated comparable performance with relatively similar R^2^ values [27]. More efforts are still being devoted towards using machine learning algorithms to predict mechanical properties of different types of concrete, for example, in their recent work, Shang et al. [28] demonstrated how machine learning models including decision tree and AdaBoost were utilized to predict the compressive strength and splitting tensile strength of concrete containing recycled coarse aggregate (RCA), reaching high R^2^ values.

In addition to regression models, classification models have also been used in the field of concrete technology; for instance, Akpinar and Khashman [29] used ANN to successfully classify compressive strength grade of different concrete mixes as low, normal or high strength. Further, Hilal Erdal [30] used two-level and hybrid ensembles of decision trees for predicting high performance concrete compressive strength.

Despite these efforts, classification of high strength concrete mixes based on their ingredients is still an open issue. Thus, in this work, we test the hypothesis that given enough training data, a trained machine learning model can accurately classify mix design methods based on mix proportions. Specifically, a decision tree model will be used to classify high strength concrete mix design methods based on their produced concrete proportions, namely, the amounts of cement, water, gravel, sand, and chemical admixtures. The following section discusses the machine learning model used in this work, i.e., decision trees.

### 1.2. Decision Trees

Decision tree models [31] are a group of algorithms that can detect classes within a dataset by passing information along a decision tree nodes and branches starting from the root node to the leaf nodes which contain the predicted class.

**Figure 1:**
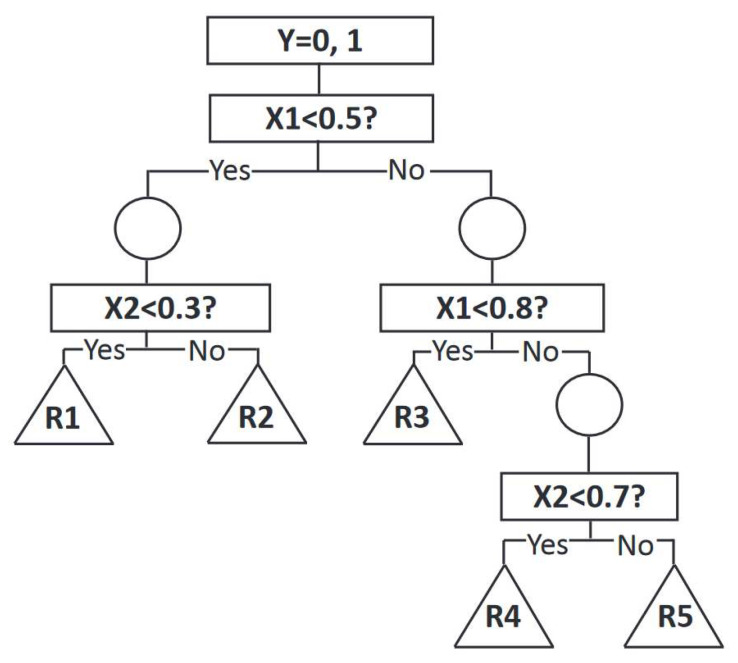
An example of a decision tree that is based on binary target variable Y (adapted from [32]).

The example tree in Figure 1 shows a binary target variable is classified to either Y = 0 or Y = 1, based on two predictors, X1 and X2, whose values range from 0 to 1. Nodes and branches constitute the essential components of a decision tree model. During the development of the decision tree, three processes take place, splitting, stopping, and pruning [32]. There exist many algorithms that implement decision trees, such as classification and regression trees (CART) [31], C4.5 [33], Chi-squared automatic interaction detection (CHAID), and QUEST, which is the abbreviation of quick, unbiased, efficient, statistical tree [34].

The rest of the paper is organized as follows: methods are described in Section 2, results are presented and discussed in Section 3, and conclusions are presented in Section 4. Further, a paragraph describing the limitations of this work is included at the end of the paper.

## 2. Materials and Methods

### 2.1. Dataset

Data is considered to be the backbone of any machine learning model; in this work, thousands of mix designs were generated and used to train a decision tree model. As designing thousands of concrete mixes to train the model is a tiresome process, computer programs that implement high strength concrete mix design methods were used. Particularly, MATLAB (MathWorks, Inc., Natick, MA, USA) was used to develop high strength concrete mix design programs to generate the dataset for the machine learning model. The developed programs design high strength concrete mixes using three mix design methods [35]. Outputs of the developed programs were compared to manual calculations to make sure no discrepancies exist between computer programs outputs and manual calculations. A total of 10 comparisons between the output of the programs and manual calculations were performed for each method. A representative comparison example for each method is shown in Table 1, Table 2 and Table 3. A total of 1000 high strength concrete mix designs were generated by the programs for each method, with a total of 3000 mix designs for training and testing purposes. All concrete mixes in the dataset were designed by the programs to produce concrete mixes of 28-day compressive strengths no less than 60 MPa and no greater than 82 MPa, cylinder strength for both ACI and Aïtcin methods and cube strength for modified DOE method. The inputs and outputs of each mix design program are summarized below:

#### 2.1.1. ACI 211.4R-08

Input: The required strength, material properties (specific gravities, bulk densities, and moisture content), maximum nominal size of coarse aggregates, required workability (slump), whether fly ash and/or admixtures (HRWRA) are used, cost of concrete constituents, casting quantity, and whether previous test records are available.

Output: Weights (per cubic meter and per provided casting quantity) of cement, water, fine and coarse aggregates as well as fly ash, admixtures (HRWRA), and the associated cost.

#### 2.1.2. Aïtcin Method

Input: The required strength, material properties, properties of superplasticizer, aggregate shape, whether slag or/and silica fumes and/or fly ash are used, moisture content of fine and coarse aggregates, cost of concrete constituents, casting quantity, and whether previous test records are available.

Output: Weights (per cubic meter and per provided casting quantity) of cement, water, fine and coarse aggregates as well as fly ash, silica fume, slag cement, superplasticizers, the calculated water/cement ratio, and the associated cost.

#### 2.1.3. Modified DOE

Input: The required strength, material properties, maximum cement content, types of coarse and fine aggregates, type of cement, cost of concrete constituents, casting quantity and whether previous test records are available.

Output: Weights (per cubic meter and per provided casting quantity) of cement, water, fine and coarse aggregates as well as superplasticizers (HRWRA), suggested ratios for coarse aggregate and the associated cost.

### 2.2. Visualizing the Dataset

**Figure 2:**
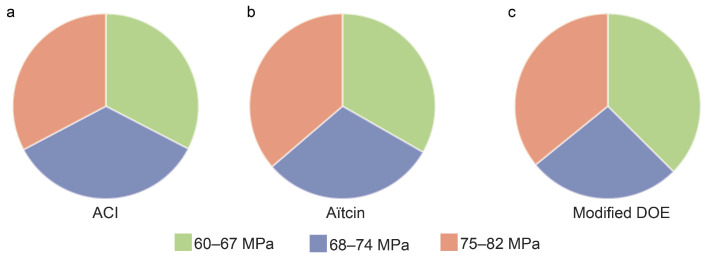
Training data produced by the mix design programs showing uniformity in distribution of compressive strength in the three classes: (**a**) ACI, (**b**) Aïtcin, and (**c**) Modified DOE.

**Figure 3:**
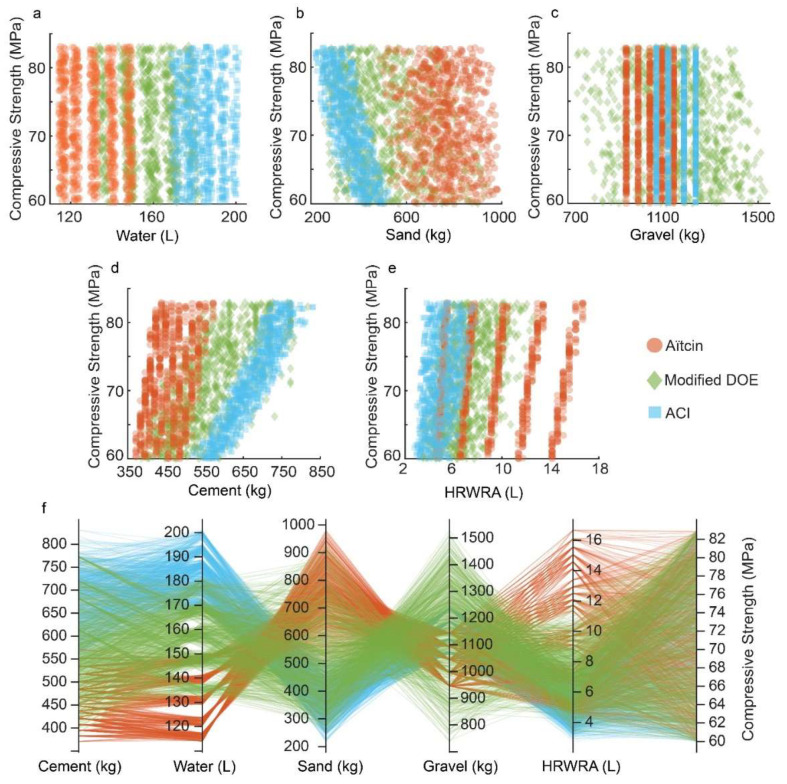
Visualizing the training data. (**a**) Compressive strength vs. water. (**b**) Compressive strength vs. sand. (**c**) Compressive strength vs. gravel. (**d**) Compressive strength vs. cement. (**e**) Compressive strength vs. HRWRA. (**f**) Shows the intertwinement of mix proportion when overlapped, making the determination of the mix design method based on mix ingredients a difficult task.

Figure 2 shows distribution of strengths of the concrete mixes produced from each method. Pie charts show that for each mix design method, the designed mixes contain uniformly distributed strengths. The relationships between mix ingredients and strength are not always clear, and intricate overlaps are present between design methods, making it a very challenging task to distinguish the design method based on mix proportions for a specified compressive strength, see Figure 3a–e. This difficulty can be clearly observed when parallel lines of all mixes produced by all three methods are overlapped, see Figure 3f. The overlap that exists between the paths leading to the required strength clearly shows how it is very difficult to tell which method was used to design which mix, hence necessitating a machine learning algorithm to tell them apart.

### 2.3. Features

The tree models make use of data features to be trained and fitted. In this work, the features used to train the model were the concrete mix proportions per one cubic meter of each mix design and the corresponding compressive strength. In particular, the amounts of cement in kg, water in liter, sand in kg, gravel in kg and HRWRA in liter, as well as compressive strength in MPa.

### 2.4. Coding Environment

MATLAB (MathWorks, Inc., Natick, MA, USA) was used to preprocess and visualize the dataset, as well as train and test the model.

### 2.5. Preprocessing of Dataset

Before training the dataset, it was standardized using mean and standard deviation.

### 2.6. Splitting the Dataset

The dataset (3000 mix designs) were split into training (2400 mix designs) and testing (600 mix designs).

### 2.7. Model Choices

Three types of decisions trees were used, according to the number of nodes in the tree, namely, fine, medium, and coarse trees. Further, during model training, 5-fold cross validation was used to prevent overfitting. For fine tree models, the number of maximum splits was specified to be 100 splits and the splitting criterion to be Gini’s Diversity Index [36].

### 2.8. Methods of Evaluating Classifier’s Performance

To evaluate the performance of the decision tree model, accuracy measure was used, which is defined for binary classification as the ratio of the correct predictions (true positive *TP* and true negative *TN*) to the total number of predictions (true positive *TP*, true negative *TN*, false positive *FP*, and false negative *FN*):

(1) $$Accuracy=\frac{TP+TN}{TP+TN+FP+FN}$$

In addition to accuracy, the receiver-operating characteristic (ROC) was used, which shows true positive rate versus false positive rate for the tree classifier. It is usually used for evaluating binary classification performance but can be used in multi-class classification, as is the case in this work, by evaluating one-vs-rest. A perfect classifier is one that correctly classifies all data to their actual classes; this perfect classifier appears in the ROC as a right-angle curve whose right angle is in the upper left-corner portion of the curve. ROC shows a poor classifier as a curve close to a line inclined at 45 degrees. The area under the ROC (AUC) is a sign of the performance of the classifier; a perfect classifier will have an AUC of 1.

Confusion matrix was also used to further evaluate the tree classifier, which is a matrix whose rows correspond to the predicted class, and columns correspond to the true class. The diagonal of the confusion matrix shows instances that were correctly classified and the off-diagonal shows instances that were incorrectly classified.

### 2.9. Feature Importance

A machine learning model shall be efficient as well as accurate; therefore, the number of features used to train the model shall be optimized. This can be performed by using principal component analysis (PCA) [37], and minimum redundancy maximum relevance (MRMR) [38].

#### 2.9.1. Principal Component Analysis (PCA)

PCA reduces the dimensionality of the model by linearly transforming predictors, removing redundant predictors, and keeping only principal predictors [37].

#### 2.9.2. Minimum Redundancy Maximum Relevance (MRMR)

MRMR algorithm determines the importance of each feature in the dataset to the classification process. The MRMR algorithm’s goal is to find an optimal set of features whose relevance to outcome variable is maximum and whose redundancy is minimum [38].

## 3. Results and Discussion

After all models were fitted to training data, they were tested using the testing dataset, which contains 20% of the entire dataset i.e., 600 mix designs. The classification accuracies of all models on training data and testing data are summarized in Table 4, below.

From the accuracy values presented in Table 4, it can be said that the simple decision tree model successfully solved the mix design method classification problem with high accuracy. Such high accuracies are guaranteed not to come from overfitting the training dataset, because a 5-fold cross validation was used in the training phase to prevent overfitting. In addition, when the trained models are tested using previously unseen data (testing dataset) they show high accuracy, indicating great generalizability potential.

**Figure 4:**
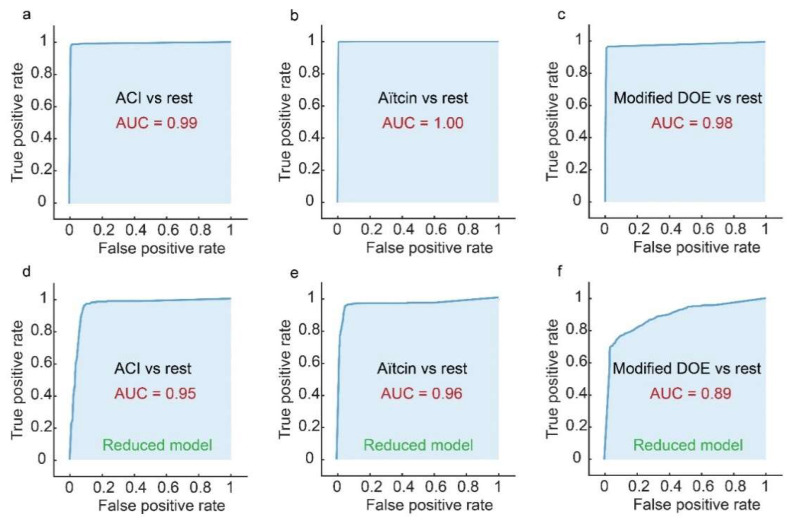
Evaluating the performance of the tree classifier on training data. (**a**) ROC curve of ACI method versus Aïtcin and modified DOE. (**b**) Aïtcin method versus ACI and modified DOE. (**c**) Modified DOE method versus ACI and Aïtcin. Similar ROC curves are shown in (**d**–**f**), however, for the case when reduced-dimensionality tree models are used.

Figure 4a presents ROC curves, which show the performance of the tree classifier on training data. ROC curve of ACI-vs-rest, with ACI assigned as the positive class and the other methods assigned as the negative class shows that the AUC is 0.99, with a true positive rate (TPR) of more than 0.98. These numbers indicate high classification performance, because a true positive rate of above 0.98 indicates that the used model correctly classifies more than 98% of the instances to the true class.

In Figure 4b, Aïtcin method was assigned to be the positive class and the other methods to be the negative class. In this case, the AUC was found to be 1.00, which indicates that the classifier has perfect classification performance for this class. In Figure 4c, modified DOE was assigned to be the positive class and the other methods to be the negative class. In this case, the area under the curve was found to be 0.98, which signifies high classification performance for this class.

**Figure 5:**
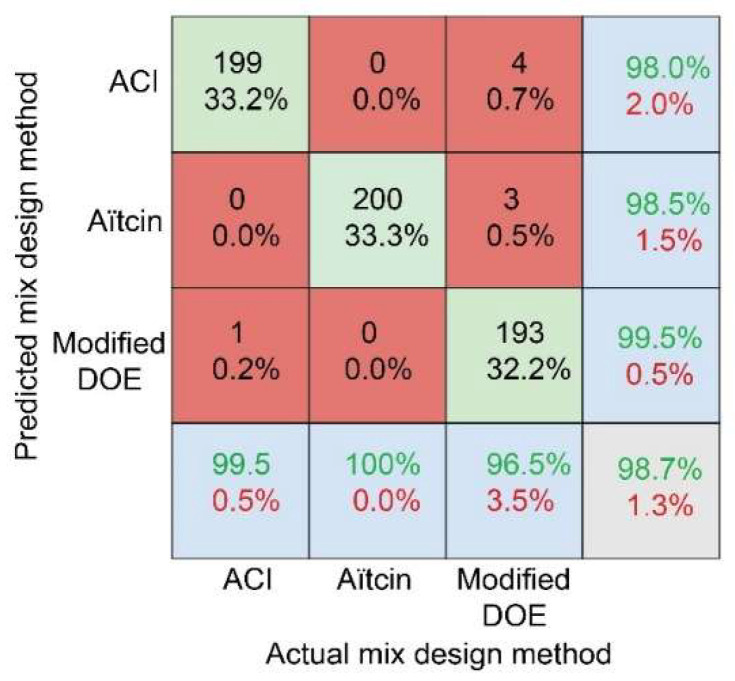
Evaluating the performance of the tree classifier using confusion matrix on testing data.

Accuracy of the fine tree model that was tested using testing data is shown in the lower right corner in the confusion matrix shown in Figure 5. In each cell in the matrix, both the number of observations and the percentage of the number of observations are shown. The most-right column shows the percentages of all the observations that are predicted to belong to each class (ACI, Aïtcin, and modified DOE) that are both assigned correctly (precision) and incorrectly (false discovery rate). The row at the bottom of the matrix shows the percentages of all the observations that belong to each class that are correctly classified (recall) and incorrectly classified (false negative rate). Based on the confusion matrix, I claim that the tree model is an excellent classifier, achieving an accuracy of 98.7%, a precision of at least 98%, and a recall of at least 96.5%, across all classes when evaluated using testing data.

**Figure 6:**
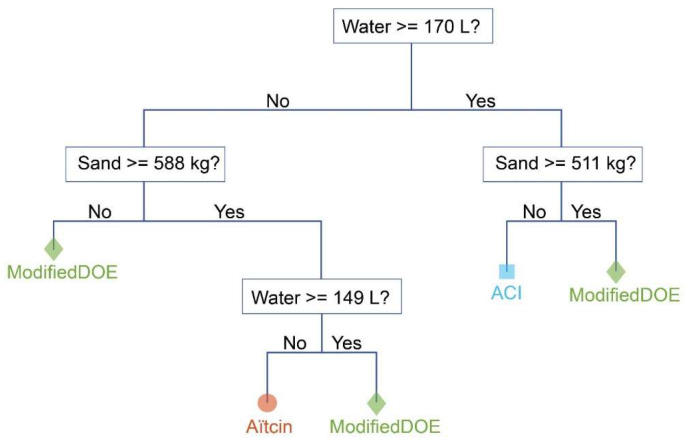
The trained coarse tree classifier; this simple model achieved a testing accuracy of 88.3%.

The trained tree model can be viewed it its actual form, however, I only show the coarse tree model herein because the fine tree and the medium tree models are too dense to be interpreted properly. The coarse tree classifier is shown in Figure 6.

**Figure 7:**
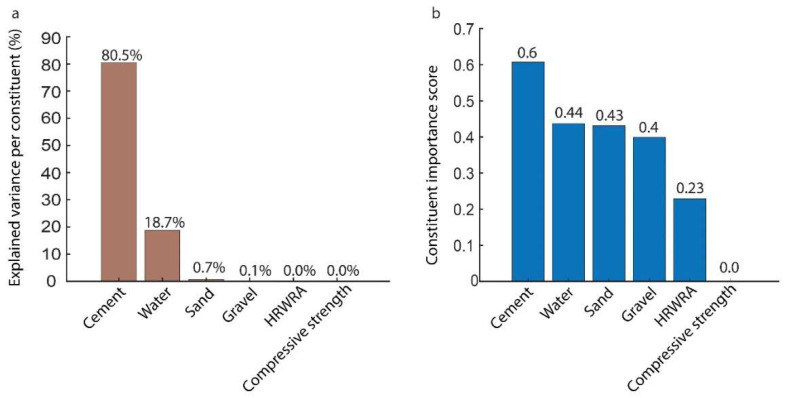
Feature importance analysis results: (**a**) Using PCA and (**b**) using MRMR.

The fine tree classifier was re-trained, however, with enabling principal component analysis this time around, to determine feature importance. The PCA-enabled fine tree classifier kept two features, which can explain 95% variance. Explained variance per feature was found to be the amount of cement: 80.5%, amount of water: 18.7%, amount of sand: 0.7%, amount of gravel: 0.1%, and 0.0% for both the amount of HRWRA and concrete’s compressive strength. Results of PCA is shown in Figure 7a.

Degree of importance of each feature was determined using the MRMR algorithm, which assigns scores to each feature, indicating its importance. The amount of cement was found by MRMR to be the most important feature, with a score of 0.6, followed by the amount of water: 0.44, amount of sand: 0.43, gravel: 0.4, HRWRA: 0.23 and compressive strength: 0.0. The drop in score between the amount of cement and amount of water is relatively large, which emphasizes that cement is the most important predictor of the mix design method. On the other hand, the amount of gravel and compressive strength were found to be weak predictors of the mix design method. The results of MRMR is shown in Figure 7b.

Based on the results of the PCA analysis, only the two most important features (amount of cement and amount of water) were used in fitting reduced tree models. As can be observed in Table 5, accuracies of the reduced models were less than those of original models with the full list of features.

In concrete technology, we seldom talk about the amount of cement and amount of water separately; instead, we use the water/cement ratio or (W/C) for short. It can be speculated that the W/C can be of importance in the determination of mix design method; however, as it is merely a linear combination of two features, then its information is already embedded in the training dataset and hence its contribution to the outcome variable may be limited. In addition, the current set of features aided in classifying the mix design methods with very high accuracy, eliminating the need for more features.

After only using principal predictors (amounts of cement and water) in training the tree model, ROC was used to test the performance of the reduced models. Figure 4d presents ROC curves, which show the performance of the reduced tree classifier on training data. ROC curve of ACI-vs-rest, with ACI assigned as the positive class and the other methods assigned as the negative class, shows that the AUC for the reduced model is 0.95, which indicates that the classification performance was slightly affected by the omittance of some of the features. In the case where Aïtcin method was assigned to be the positive class and the other methods to be the negative class, Figure 4b, the AUC was found to be 0.96, which indicates that the performance of the classifier was also slightly affected by the omittance of some of the features. For the case where modified DOE was assigned to be the positive class and the other methods to be the negative class, the classification performance was greatly affected by the omittance of some of the features with an area under the curve of 0.89.

### Limitations

While this study successfully developed a model that is capable of classifying mix design methods with very high accuracies, a few shortcomings exist. For example, the training data used in training the decision tree model are synthesized. This approach works perfectly, provided that the proportions of the mix are exactly per the mix design recommendations, before any field adjustments. However, sometimes field conditions necessitate that concrete consistency is adjusted, which distorts original mix design. Hence, it is necessary that the developed model is trained/tested using real experimental data (training/testing the model using field-adjusted concrete proportions). Another limitation is that only one machine learning model is used in this work, while many more models can be tested and compared to determine the most accurate and efficient model.

## 4. Conclusions

Mix design methods are used by concrete manufacturers all over the world to design and make concrete mixes according to the customers’ needs. It is of great importance that site/construction engineers know the method by which a concrete mix was designed, so that they can correctly adjust concrete mixes according to field conditions, interpret fresh concrete tests results, as well as satisfy quality control requirements. However, determining the method by which a concrete mix was designed can be a difficult task because design methods usually suggest similar mix proportions for certain required properties. This work solved this classification problem via the use of machine learning. In particular, this work achieved the following:

- Machine learning, specifically decision tree models were trained to classify high strength concrete mix design methods based on concrete mix proportions with high accuracy. It was shown that knowledge of the basic amounts of the basic ingredients of high strength concrete mix is enough for the model to accurately determine the mix method by which it was designed.
- Feature importance analyses demonstrated that the amount of cement and water in the concrete mix are the most important predictors of the used mix design method.
- In this work, a novel high-accuracy model for determining the mix design method, based only on mix proportion, was presented.

Future work includes training and testing machine learning models using real experimental data (training/testing the model using field-adjusted concrete proportions). Further, the author intends to experiment with more machine learning models to determine the most accurate and efficient model for classifying mix design methods.

## Figures and Tables

**Table 1:** A comparison between manual calculations and program outputs for ACI211.4R-8 method [35].

Mix Proportions	Manual	Program	Variation	
			Numeric	%
Cement (kg/m^3^)	334.8	337.94	−3.14	−0.93
Water (kg/m^3^)	188.92	188.89	0.03	0.02
FA (kg/m^3^)	613.4	610.09	3.31	0.54
CA (kg/m^3^)	1072.5	1072.5	0	0.00
Fly ash (kg/m^3^)	63.77	64.37	−0.6	−0.93
Superplasticizer (kg/m^3^)	2.39	2.41	−0.02	−0.83

**Table 2:** A comparison between manual calculations and program outputs for Aïtcin method [35].

Mix Proportions	Manual	Program	Variation	
			Numeric	%
Cement (kg/m^3^)	439.015	442	−2.99	−0.68
Water (kg/m^3^)	118.114	117.98	0.13	0.11
FA (kg/m^3^)	654.836	658.61	−3.77	−0.58
CA (kg/m^3^)	1089	1089	0	0
Silica Fume (kg/m^3^)	25.824	26	−0.18	−0.68
Fly ash (kg/m^3^)	51.649	52	−0.35	−0.68
Superplasticizer (kg/m^3^)	7.65	7.7	−0.05	−0.65

**Table 3:** A comparison between manual calculations and program outputs for modified DOE method [35].

Mix Proportions	Manual	Program	Variation	
			Numeric	%
Cement (kg/m^3^)	617.28	614.49	2.79	0.45
Water (kg/m^3^)	170.26	170.31	−0.05	−0.03
FA (kg/m^3^)	518.66	517.61	1.05	0.2
CA (kg/m^3^)	1098.54	1106.28	−7.74	−0.7
Superplasticizer (kg/m^3^)	6.688	6.66	0.03	0.42

**Table 4:** Accuracy of tree classifiers.

Model	Training Accuracy	Testing Accuracy
Decision trees: Fine	98.2%	98.7%
Decision trees: Medium	97.9%	97.8%
Decision trees: Coarse	90.5%	88.3%

**Table 5:** Classification accuracy of reduced-dimensionality classifiers.

Model	Training Accuracy	Testing Accuracy
Reduced-dimensionality fine decision tree	86.3%	86.5%
Reduced-dimensionality medium decision tree	85.1%	85.3%
Reduced-dimensionality coarse decision tree	81.3%	81.3%

## Data Availability

The data presented in this article are available upon reasonable request.
